# Alice in Wonderland and Ekbom Syndromes in a Bipolar I Manic Episode: A Case Report With Neuroimaging Findings

**DOI:** 10.1155/crps/1065938

**Published:** 2026-04-07

**Authors:** Yuta Hori, Satoru Yanagaki, Masashi Nibuya, Seishu Nakagawa, Eiji Suzuki

**Affiliations:** ^1^ Division of Psychiatry, Tohoku Medical and Pharmaceutical University, Sendai City, Miyagi, Japan, tohoku-mpu.ac.jp; ^2^ Division of Radiology, Tohoku Medical and Pharmaceutical University, Sendai City, Miyagi, Japan, tohoku-mpu.ac.jp

**Keywords:** Alice in Wonderland syndrome (AIWS), bipolar I disorder, brain single-photon emission computed tomography (SPECT), case report, Ekbom syndrome, manic episode

## Abstract

With recent advances in anatomical and functional brain mapping, Alice in Wonderland syndrome (AIWS), a perceptual distortion disorder, has received increased attention. We report the case of a 67‐year‐old man with bipolar I disorder (manic episode), AIWS, and delusional parasitosis (Ekbom syndrome). The patient exhibited diverse perceptual distortions across visual, auditory, and tactile modalities. In addition to derealization and depersonalization, he showed a distorted sense of time. Single‐photon emission computed tomography (SPECT) revealed a transient but significant decrease in blood flow in the right posterior cingulate region, accompanied by relatively increased blood flow in the bilateral occipital regions during the episode.

## 1. Introduction

Metamorphic visual perception of one’s own body and the external environment, with or without a distorted sense of time, has been described as Alice in Wonderland syndrome (AIWS), named after Carroll’s [1] Alice’s Adventures in Wonderland. Fewer than 200 cases have been reported in the medical literature since the first description by Lippman [2] in 1952 and the second by Todd [3] in 1955, as systematically reviewed by Blom [4] in 2016. AIWS is not listed in the Diagnostic and Statistical Manual of Mental Disorders, Fifth Edition (DSM‐5, American Psychiatric Association). To establish the diagnostic criteria for perceptual disorders, it is essential to accumulate case reports that include functional neuroimaging analyses. We present a case of a patient with bipolar I disorder, manic episode, who exhibited diverse perceptual distortions. Single‐photon emission computed tomography (SPECT) revealed a transient but significant hypoperfusion in the right posterior cingulate region during the AIWS episode.

## 2. Case Presentation

Mr. A, a 67‐year‐old man, was admitted to the psychiatric unit of our university hospital for his first manic episode. He had been diagnosed with depression 22 years earlier at a psychiatric clinic. His most recent prescription included maprotiline (30 mg/day) and brotizolam (0.25 mg/day) for insomnia, which had remained unchanged for many years. He had no major physical comorbidities aside from mild hypertension and benign prostatic hyperplasia. There was no history of substance abuse, migraine, epilepsy, or head injury, and he had abstained from alcohol for several years.

The manic and perceptual symptoms appeared simultaneously. For 1 month, he exhibited multiple characteristic disturbances, including altered perception, derealization, and depersonalization, in addition to classic manic symptoms, such as talkativeness, hyperactivity, reduced need for sleep, irritability with violent behaviors, flight of ideas, anxiety, agitation, and distractibility. These manic features persisted for about 2 months. He was admitted to our psychiatric unit 2 weeks after the initiation of the symptoms. He also developed delusional parasitosis with visual hallucinations of numerous small bugs crawling over his whole body (Ekbom syndrome; reviewed by Mindru et al. [5]). He reported unbearable cutaneous itching and compulsively washed his bedclothes and curtains, and repeatedly cleaned windows and doors throughout the day and night. He described visual distortions consistent with AIWS as follows: “I, others, and things around me are getting taller and bigger in height and volume, and my wife seems to be rapidly decreasing in size.” Additional visual metamorphosias included perceptions that the faces of others were approaching him, the shape of a table‐tennis ball was changing, and the faces of a white dog and his family members turned completely black. Time distortion (reviewed by Blom JD et al. [6]) was expressed as “Other people’s speech is heard louder and at accelerated speed. Time is passing much faster, and cars on television are moving more rapidly.” Derealization and depersonalization (reviewed by Hunter et al. [7]) were reflected in his statements such as: “The television reported that our national table‐tennis team won a world championship tournament, but this is impossible and cannot be real.” To confirm his identity, he repeatedly telephoned his friends and even called his own home. He also many times checked his car license card and a copy of his family registry to reassure himself of his name.

**Figure 1:**
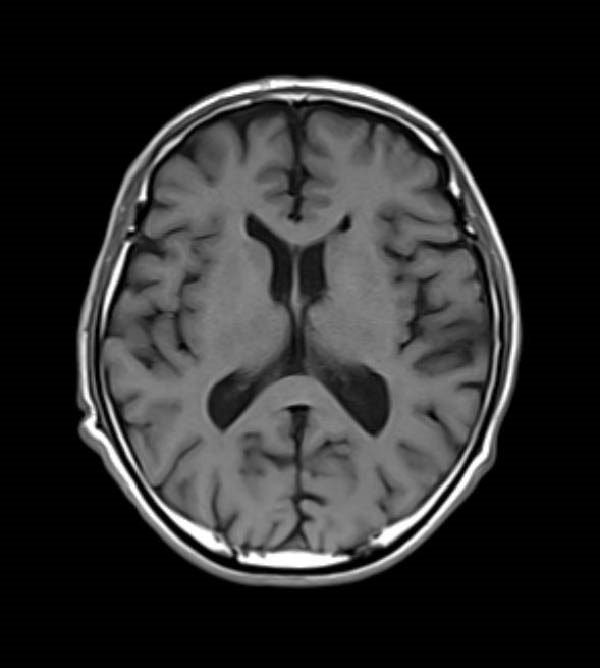
Magnetic resonance imaging (MRI) revealed no significant brain atrophy. MRI was performed with a 1.5‐T scanner (Siemens Healthineers) using a T1‐weighted sequence with the following parameters: repetition time = 450 ms; echo time, 12 ms; field of view, 207 × 229 mm²; matrix size, 319 × 288; slice thickness = 5.0 mm.

Laboratory tests revealed no abnormalities in liver, kidney, thyroid, lipid, or glucose metabolism. No fever was noted before or after symptom exacerbation, and blood examination results showed no elevations in C‐reactive protein, white blood cells, or lymphocytes. Magnetic resonance imaging (MRI) showed no significant atrophy (Figure 1), and magnetic resonance angiography (MRA) demonstrated no evidence of cerebral artery stenosis nor dissection.

A SPECT brain scan using N‐isopropyl‐p‐[^123^I] iodoamphetamine (^123^I‐IMP) was performed on the 7th day after admission. Voxel‐based statistical analysis revealed significant hypoperfusion in the left parietal cortex (*Z* score = –2.24 SD compared with age‐matched controls), the right precuneus (–2.53 SD), and the right posterior cingulate region (–1.72 SD; Figure 2A,B). Hyperperfusion was detected in the occipital regions but did not reach statistical significance. The values were +0.74 SD/+0.70 SD in the right/left lateral occipital regions and +0.81 SD/+0.83 SD in the right/left medial occipital regions, below the significance threshold of *Z* = +1.64 SD; Figure 2A,B). Two months after admission, hypoperfusion improved to –1.93 SD in the left parietal cortex, –1.84 SD in the right precuneus, and –0.17 SD in the right posterior cingulate region. In addition, right parietal hypoperfusion (–1.28 SD; not significant) shifted toward slight hyperperfusion (+0.25 SD). No obvious changes were observed in the relative hyperperfusion of the bilateral occipital regions 2 months after admission (Figure 3A,B). SPECT images were acquired 15 min after intravenous injection of ^123^I‐IMP (118 MBq) using a gamma camera (NM/CT 870 DR, GE Healthcare) equipped with extended low‐energy general‐purpose collimators. Images were obtained in a 128 × 128 matrix during continuous rotation (six rotations of 5 min each).

Figure 2(A) A single‐photon emission computed tomography (SPECT) brain scan using N‐isopropyl‐p‐[^123^I] iodoamphetamine (^123^I‐IMP) performed on the 7th day after admission revealed significantly reduced uptake in the left parietal cortex (gray arrow; *Z* = –2.24 SD compared with age‐matched controls), right precuneus (short white arrow; *Z* = –2.53 SD), and the right posterior cingulate region (long white arrow; *Z* = –1.72 SD). Relative hypoperfusion was also observed in the right parietal cortex (medium white arrow; *Z* = –1.28 SD). Areas shown in red color indicate hypoperfusion in (A) and Figure 3A. (B) Relative hyperperfusion in the bilateral occipital regions was not significant (white and gray arrows indicate the right and left occipital regions, respectively). Areas shown in red color indicate hyperperfusion in (B) and Figure 3B.

**Figure:**
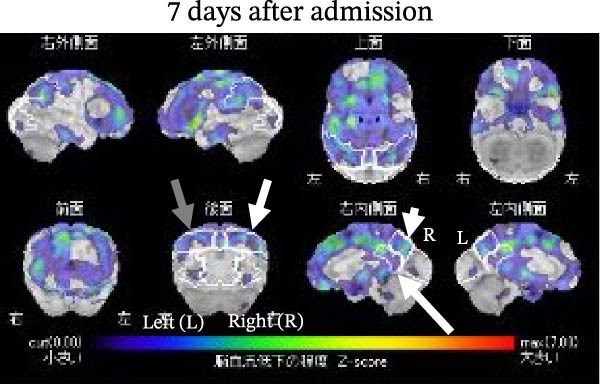
(A)

**Figure:**
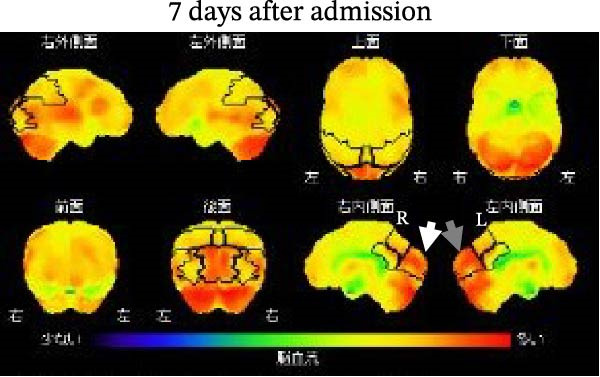
(B)

Figure 3(A) Two months after admission, hypoperfusion improved in the right precuneus (*Z* = –1.84 SD), the right parietal cortex (*Z* = +0.25 SD), and the right posterior cingulate region (*Z* = –0.17 SD). No appreciable change was observed in the left parietal cortex. (B) No obvious alterations were observed in the bilateral occipital regions.

**Figure:**
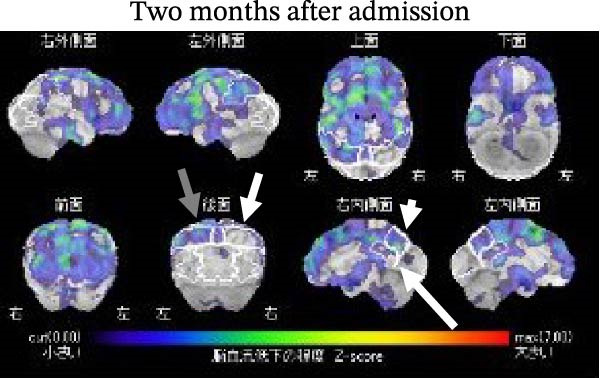
(A)

**Figure:**
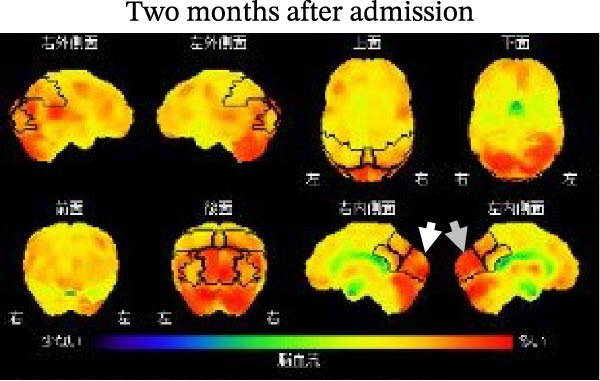
(B)

Psychotropic treatment for manic symptoms included oral valproic acid (600 mg/day; serum concentration, 78.9 μg/mL) and olanzapine (12.5 mg/day). Although he was able to communicate normally with family members and others, his Mini‐Mental State Examination (MMSE) score was 20/30, indicating moderate cognitive impairment, particularly in calculation and recall. To evaluate cognitive impairment, the Japanese version of the MMSE from Nihon Bunka Kagakusha Co. Ltd. was used, and permission to cite the data of MMSE in the present text was obtained from Psychological Assessment Resources, Inc., Florida, U.S.A.

To ensure the quality of the present manuscript, this case report was prepared in accordance with the 2013 CARE checklist [8], and the 13‐item list is included in the Supporting Information 1.

## 3. Discussion

AIWS is a rare perceptual disorder characterized by visual metamorphopsias and temporal distortions, sometimes accompanied by depersonalization and derealization [4]. In the present case, a series of typical AIWS symptoms was observed, including macropsia (seeing objects larger), micropsia (seeing objects smaller), total‐body macrosomatognosia (perceiving one’s own body as bigger than it is), pelopsia (perceiving objects as closer), dysmorphopsia (shape distortion, such as a table‐tennis ball changing form), kinetopsia (illusionary movement), dyschromatopsia (color confusion), the quick‐motion phenomenon (accelerated psychological time), derealization, and depersonalization. In addition to frequently and temporally observed symptoms as aura of migraine patients [9], AIWS has most commonly been reported in patients with substance intoxication, encephalitis, and brain lesions [4]. To date, three cases of AIWS have been reported in men with major depressive disorder [10–12] and one case in a woman with a depressive episode of bipolar disorder associated with lithium‐induced hyperparathyroidism [13]. To our knowledge, this is the first reported case of AIWS emerging simultaneously with a bipolar I manic episode.

Although delusional parasitosis is not categorized as a sensory abnormality, it appeared to occur secondarily in this patient, likely due to tactile hypersensitivity and enhanced itch perception. This is the first documented report of comorbid delusional parasitosis in a patient with AIWS. Considering that the sense of time is sometimes regarded as a sensory modality [14], this patient exhibited abnormal perceptions across the visual, tactile, auditory, and temporal domains.

Derealization and depersonalization are conceptually distinct from altered perception, yet comorbidities with AIWS and bipolar disorder are not rare. A systematic review reported that derealization occurred in 10% (17/169) of AIWS cases and depersonalization in 4% (7/169) [4]. In addition, a comorbidity survey in the United States indicated prevalence of clinically significant depersonalization/derealization in bipolar I disorder (8.3%) and bipolar II disorder (11.1%) [15]. Although it is difficult to discriminate the etiology of derealization and depersonalization in the present case from AIWS or from a mood disorder, fragile self‐continuity in individuals with distorted time perception [16] has been suggested as an important factor in understanding derealization and depersonalization.

A review of lesion‐mapping studies examining 30 adult cases of lesion‐associated AIWS using CT and/or MRI reported maximum spatial overlap in the right occipital cortex [17, 18]. Another review using lesion‐network mapping of 37 adult lesion‐induced AIWS cases identified common lesions with shared connectivity between the right extrastriate body area in the occipital cortex and the inferior parietal cortex [19]. The hypoperfusion observed in the right precuneus, parietal region, and posterior cingulate regions in the present case, areas functionally connected to the right occipital cortex, is consistent with these findings.

A functional MRI study demonstrated increased connectivity between the lateral occipital region and the posterior superior temporal sulcus in 12 adult patients with AIWS and migraine [20]. Although SPECT studies in adult AIWS cases remain limited, individual case reports have described hypoperfusion in the right frontoparietal operculum in a 53‐year‐old woman [21] and biparietal and bitemporal hypoperfusion in a 68‐year‐old patient with Lewy body dementia and AIWS [22]. Collectively, these findings suggest that AIWS is pathophysiologically related to altered visual network connectivity involving the occipital cortex.

Furthermore, significant occipital hypermetabolism was detected by fluorodeoxyglucose positron emission tomography (FDG‐PET) in a 67‐year‐old man with depression and AIWS [12], and increased focal hyperperfusion was reported in the right occipital cuneus in a 47‐year‐old woman with aripiprazole‐induced AIWS [23]. Both reports correspond well with the present case. We propose that the improvement of hypoperfusion in the right posterior cingulate, precuneus, and parietal regions, together with persistent relative hyperperfusion in the occipital regions, supports the hypothesis of an imbalanced network involving occipital connectivity in AIWS. No consistent and significant hypoperfusion in the right posterior cingulate, parietal regions, and precuneus has been reported in mania [24, 25].

## 4. Limitations

Inconsistent perfusion changes between manic and euthymic states have been reported in the cerebral cortex. However, many studies have demonstrated lower frontoparietal cortical blood flow in patients with mania compared with healthy controls [24, 26]. The relationship between hypoperfusion in specific cortical regions and the manic state was not clarified in the present case. Relative bilateral hyperperfusion in the occipital cortex was observed, but the increase did not reach statistical significance. No brain atrophy was detected on MRI, and the impact of moderate cognitive impairment observed on the MMSE should be interpreted cautiously with continued follow‐up of the patient.

## 5. Conclusion

We report a case of bipolar I disorder with a manic episode, accompanied by AIWS and Ekbom syndrome. The influence of multiple altered sensory modalities, including visual, auditory, tactile, and temporal perception, was evident. Neuroimaging revealed a transient decrease in blood flow in the right parietal, precuneus, and posterior cingulate regions. Further accumulation of case series is required to clarify the pathophysiological mechanism of AIWS and to establish perceptual distortion disorder as a diagnostic category supported by biological evidence.

## Author Contributions

Yuta Hori participated in clinical management of the patient as a psychiatrist and contributed to the data collection and writing. Satoru Yanagaki contributed to the analyses of MRI and SPECT image data. Masashi Nibuya and Seishu Nakagawa cooperatively contributed to drafting and editing the manuscript in addition to the literature search and the interpretation of the findings. Eiji Suzuki had a role in conceptualization and supervision.

## Funding

This study did not receive any specific grant from funding agencies in the public, commercial, or nonprofit sectors.

## Consent

Written informed consent for publication of this case report was obtained from the patient and his wife. All potentially identifying information has been removed to protect patient privacy.

## Conflicts of Interest

The authors declare no conflicts of interest.

## Supporting Information

Additional supporting information can be found online in the Supporting Information section.

## Supporting information


**Supporting Information** The 13‐item 2013 CARE checklist [8] is included in the Supporting material.

## Data Availability

The MRI and SPECT datasets supporting the findings of this study are available from the corresponding author upon reasonable request.
