# Supplementary material for: Alice in Wonderland and Ekbom Syndromes in a Bipolar I Manic Episode: A Case Report With Neuroimaging Findings
Source: Case Rep Psychiatry. 2026 Apr 7;2026:1065938. doi: 10.1155/crps/1065938 (PMC13054826; doi:10.1155/crps/1065938)
Supplement: Supplementary file 1 — Supporting Information The 13‐item 2013 CARE checklist [8] is included in the Supporting material. [file CRPS-2026-1065938-s001.docx]

**2013 CARE Checklist**

1. **Title** – The diagnosis or intervention of primary focus followed by the words “case report”.

(We’ve checked our title and assured that important items are all involved in the title.)

1. **Key Words** – 2 to 5 key words that identify diagnoses or interventions in this case report (including "case report").

(In Keywords section in the text, we have listed key words including case report.)

1. **Abstract** – (structured or unstructured)
   - Introduction – What is unique about this case and what does it add to the scientific literature?
   - The patient’s main concerns and important clinical findings.
   - The primary diagnoses, interventions, and outcomes.
   - Conclusion – What are one or more “take-away” lessons from this case report?

(We consider all important items are involved in the abstract section. We tried to avoid speculative interpretations in the abstract section.)

1. **Introduction** – Briefly summarizes why this case is unique and may include medical literature references.

(We checked the manuscript and concluded that all these items are involved in the introduction section.)

1. **Patient Information**
   - De-identified patient specific information.
   - Primary concerns and symptoms of the patient.
   - Medical, family, and psychosocial history including relevant genetic information.
   - Relevant past interventions and their outcomes.

(We checked the manuscript and confirmed that all these items are involved in the case presentation section.)

1. **Clinical Findings** – Describe significant physical examination (PE) and important clinical findings.

(We checked the manuscript and confirmed that all these items are involved in the case presentation section.)

1. **Timeline** – Historical and current information from this episode of care organized as a timeline (figure or table).

(Although timeline figure is not presented in the manuscript, we checked and confirmed that enough information is involved in the case presentation section.)

1. **Diagnostic Assessment**
   - Diagnostic methods (PE, laboratory testing, imaging, surveys).
   - Diagnostic challenges.
   - Diagnosis (including other diagnoses considered).
   - Prognostic characteristics when applicable.

(We checked the manuscript and confirmed that all these items are involved in the case presentation section.)

1. **Therapeutic Intervention**
   - Types of therapeutic intervention (pharmacologic, surgical, preventive).
   - Administration of therapeutic intervention (dosage, strength, duration).
   - Changes in therapeutic interventions with explanations.

(We checked the manuscript and confirmed that all these items are involved in the case presentation section.)

1. **Follow-up and Outcomes**
   - Clinician- and patient-assessed outcomes if available.
   - Important follow-up diagnostic and other test results.
   - Intervention adherence and tolerability. (How was this assessed?)
   - Adverse and unanticipated events.

(We reported the present case at the end of the hospitalization after his complete recovery. For this reasoning clinical findings in the follow-up period is not now available.)

1. **Discussion**
   - Strengths and limitations in your approach to this case.
   - Discussion of the relevant medical literature.
   - The rationale for your conclusions.
   - The primary “take-away” lessons from this case report (without references) in a one paragraph conclusion.

(We consider all important items indicated above are involved in the discussion, limitations, and conclusion sections of our text.)

1. **Patient Perspective** – The patient should share their perspective on the treatment(s) they received.

(In the treatment we always explained our treatments carefully and the patient had a willingness to participate in the treatments.)

1. **Informed Consent** – The patient should give informed consent. (Provide if requested.)

The patient and his family gave us written informed consents for the treatment and publish the case report in an international journal as shown in the Consent for Publication section.
